# Migratory dendritic cells in skin-draining lymph nodes have nickel-binding capabilities

**DOI:** 10.1038/s41598-020-61875-6

**Published:** 2020-03-19

**Authors:** Toshinobu Kuroishi, Kanan Bando, Reiska Kumala Bakti, Gaku Ouchi, Yukinori Tanaka, Shunji Sugawara

**Affiliations:** 1 Division of Oral Immunology, Department of Oral Biology, Sendai, Japan; 2 Division of Orthodontics and Dentofacial Orthopedics, Department of Oral Health and Development Sciences, Tohoku University Graduate School of Dentistry, Sendai, 980-8575 Japan

**Keywords:** Antigen-presenting cells, Conventional dendritic cells

## Abstract

Nickel (Ni) is the most frequent metal allergen and induces Th1-dependent type-IV allergies. In local skin, epidermal Langerhans cells (LCs) and/or dermal dendritic cells (DCs) uptake antigens and migrate to draining lymph nodes (LNs). However, the subsets of antigen-presenting cells that contribute to Ni presentation have not yet been identified. In this study, we analyzed the Ni-binding capabilities of murine DCs using fluorescent metal indicator Newport Green. Elicitation of Ni allergy was assessed after intradermal (i.d.) injection of Ni-treated DCs into ear pinnae of Ni-sensitized mice. The Ni-binding capabilities of MHC class II^hi^ CD11c^int^ migratory DCs were significantly stronger than those of MHC class II^int^ CD11c^hi^ resident DCs and CD11c^int^ PDCA1^+^ MHC class II^int^ B220^+^ plasmacytoid DCs. Migratory DCs in skin-draining and mandibular LNs showed significantly stronger Ni-binding capabilities than those in mesenteric and medial iliac LNs. An i.d. injection of IL-1β induced the activation of LCs and dermal DCs with strong Ni-binding capabilities. Ni-binding LCs were detected in draining LNs after i.d. challenge with IL-1β and Ni. Moreover, an i.d. injection of Ni-treated DCs purified from skin-draining LNs elicited Ni-allergic inflammation. These results demonstrated that migratory DCs in skin-draining LNs have strong Ni-binding capabilities and elicit Ni allergy.

## Introduction

Cutaneous MHC class II^+^ professional APCs are classified into epidermal CD207 (langerin)^+^ Langerhans cells (LCs) and dermal dendritic cells (DCs)^1,2^. LCs were originally considered to be skin-resident DCs, but are now recognized as tissue-resident macrophages, the precursors of which originate from the yolk sac and fetal liver. DCs consist of conventional DCs (cDCs) and plasmacytoid DCs (pDCs). Dermal DCs have been classified into at least three subsets: XCR1^+^ cDC1, XCR1^-^ CD11b^+^ cDC2, and XCR1^-^ CD11b^−^ double negative (DN) cDCs^2^. XCR1^+^ cDC1s express CD207 and CD103. These professional APCs capture and transport local self and non-self Ags to skin-draining lymph nodes (LNs). Two subsets of DCs are detected in skin-draining LNs: MHC class II^hi^ CD11c^int^ migratory DCs and MHC class II^int^ CD11c^hi^ resident DCs. Migratory DCs have been classified into four subsets: LCs, cDC1s, cDC2s, and DNcDCs, similar to local skin. Resident DCs comprise cDC1s and cDC2s. pDCs are detected in skin-draining LNs but not in steady-state local skin.

Although there are high levels of redundancy, each APC contributes to specific immune responses. A previous study reported that LCs mainly induce Th17 responses, particularly against *Candida albicans*^3^. cDC1s have been shown to contribute to Th1 responses and cross-presentation^3,4^. cDC2s and DNcDCs contribute to Th2 responses^5,6^. Regulatory T (Treg) cells are induced by various APC subsets including epidermal LCs^7^, cDC1s in the intestinal mucosa^8^, and cDC2s in the sublingual mucosa^9^. These findings indicated that specific APCs are responsible for immune responses against specific Ags.

Metal allergies have been classified as type-IV allergies and induce allergic contact dermatitis^10,11^. Nickel (Ni) is the most frequent metal allergen. Metal allergies are considered to be mainly caused by Th1 responses, whereas others, such as NKG2D^+^ IFN-γ^+^ CD8^+^ T cells^12^, invariant NKT cells^13^, Th17 cells^14^, and Th2 cytokine thymic stromal lymphopoietin (TSLP)^15^, are also involved. Therefore, it is important to identify which APCs contribute to the presentation of metal allergens.

Human monocyte-derived DCs produce IL-12 by stimulation with Ni and IFN-γ via the p38 MAPK, NF-κB, and interferon regulatory factor-1 pathways^16^. Moreover, Ni induces IL-23 production by human monocyte-derived DCs via the Toll-like receptor 4, p38 MAPK, NF-κB, and Janus kinase/signal transducers and activators of transcription pathways, and promotes Th17 cells^17^. Ni ions have been shown to stimulate human Toll-like receptor 4, but not its murine homolog^18^. In a murine model, Ni induced the activation of NF-κB and activator protein-1 in mouse fetal skin-derived DCs^19^. Bone marrow-derived DCs were also shown to be stimulated with Ni through the MAPK pathway^20^. These findings indicate that Ni ions stimulate human and murine DCs. However, the subsets of DCs that respond to Ni have not yet been identified.

Metal ions are considered to function as haptens, which bind to self-protein(s) and induce conformational changes. Serum albumin is a well-known metal-binding protein^21^. We reported recently that the C-X-C motif chemokine CXCL4 is a novel Ni-binding protein and augments Ni allergy at the sensitization and elicitation phases^22^. However, the underlying mechanisms by which metal ions are recognized by the immune system remain unclear.

The aim of the present study was to identify which DC subsets are responsible for Ni presentation. To achieve this, we used Newport Green (NPG), a fluorescent metal probe that specifically binds to Ni ions^23^. We examined the Ni-binding capability of APCs in regional LNs and local skin tissues. We demonstrated that migratory DCs in skin-draining and mandibular LNs exhibited strong Ni-binding capabilities. An intradermal (i.d.) injection of IL-1β, an inflammatory cytokine that plays a role in Ni allergy^24^, induced the activation of epidermal LCs and dermal DCs with strong Ni-binding capabilities. Furthermore, we showed that Ni-treated DCs from skin-draining LNs elicited Ni-allergic inflammation.

## Results

### Migratory DCs in skin-draining LNs bind to Ni

**Figure 1:**
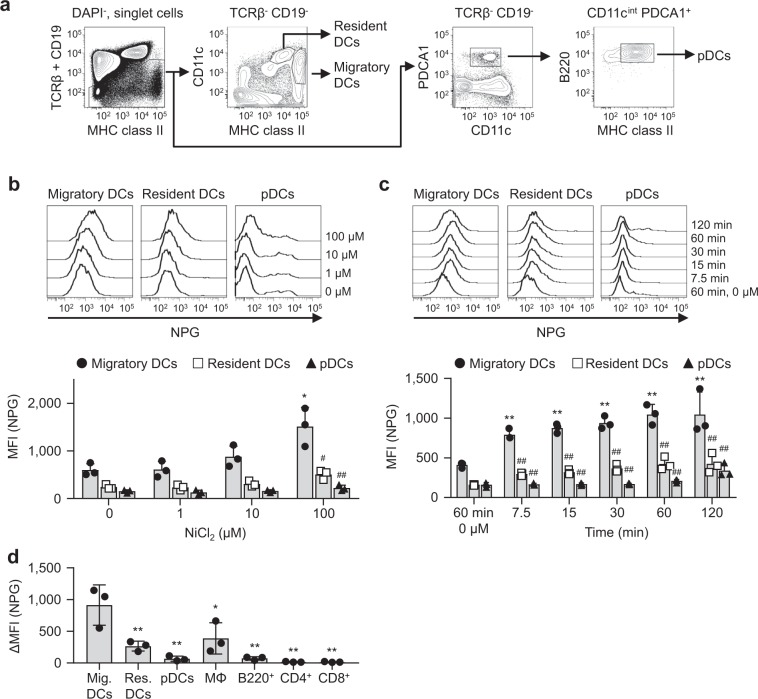
Ni-NPG staining of skin-draining LN cells. (**a**) The gating strategy used in this analysis. (**b**) Cells were incubated with the indicated concentration of NiCl_2_ for 60 min followed by NPG. Histograms represent NPG fluorescence of the indicated DCs. Results are representative of three independent experiments. Graph showing the MFI of NPG. Bars represent the mean ± SD of three independent experiments. Each symbol represents the value from an independent experiment. *P < 0.05, significantly different from the control (0 μM NiCl_2_). #*P* < 0.05, ##*P* < 0.01, significantly different from migratory DCs. (**c**) Cells were incubated with 100 μM NiCl_2_ for the indicated times followed by NPG. Histograms represent the NPG fluorescence of the indicated DCs. Results are representative of three independent experiments. Graph showing the MFI of NPG. Bars represent the mean ± SD of three independent experiments. Each symbol represents the value from an independent experiment. **P < 0.01, significantly different from the control (60 min, 0 μM NiCl_2_). ##*P* < 0.01, significantly different from migratory DCs. (**d**) Cells were incubated with 100 μM NiCl_2_ for 60 min followed by NPG. Graph showing the ΔMFI. Bars represent the mean ± SD of three independent experiments. Each symbol represents the value from an independent experiment. *P < 0.05, **P < 0.01, significantly different from migratory DCs.

We assessed the Ni-binding capabilities of DCs in skin-draining LNs. Skin-draining LN cells contained three DC subsets: MHC class II^hi^ CD11c^int^ migratory DCs, MHC class II^int^ CD11c^hi^ resident DCs, and CD11c^int^ PDCA1^+^ MHC class II^int^ B220^+^ pDCs (Fig. 1a). LN cells were incubated with various concentration of Ni for 60 min, then stained with NPG. The mean fluorescence intensity (MFI) of NPG increased in a Ni dose-dependent manner (Fig. 1b). Migratory DCs showed significantly higher MFI with 100 μM Ni than those of control (0 μM Ni). When LN cells were incubated with 100 μM Ni for the indicated time, MFI gradually increased in an incubation time-dependent manner and reached plateau at 60 min (Fig. 1c). The MFI was significantly higher in migratory DCs than in resident DCs and pDCs, regardless of Ni concentrations or incubation time. On the other hand, macrophages, B220^+^ B cells, CD4^+^ and CD8^+^ T cells exhibited significantly weaker Ni-binding capabilities than those of the migratory DCs (Fig. 1d and Supplementary Fig. S1). These results indicated that migratory DCs, but not resident DCs or pDCs specifically bound to Ni. Incubation with 100 μM Ni for 60 min was used, and Ni-binding capabilities were shown as ΔMFI, calculated as [MFI^Ni 100 μM^ – MFI^Ni 0 μM^] in subsequent experiments.

**Figure 2:**
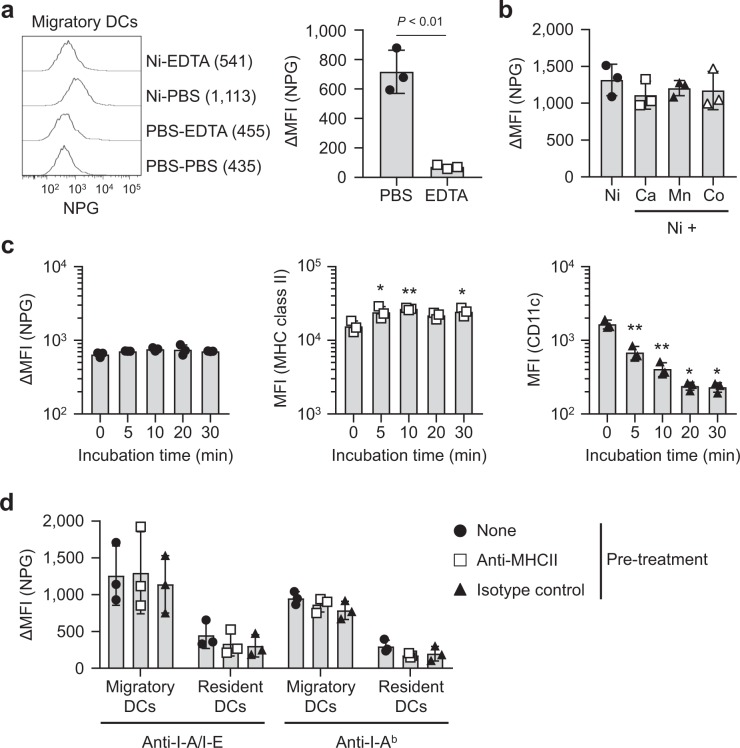
Biochemical properties of Ni-binding capabilities. (**a**) Skin-draining LN cells were incubated with 100 μM of NiCl_2_ for 60 min followed by 1 mM EDTA for 30 min. Histograms represent the NPG fluorescence of migratory DCs. Numbers shown in parentheses are the MFI of each histogram. Results are representative of three independent experiments. Graph showing ΔMFI. Bars represent the mean ± SD of three independent experiments. Each symbol represents the value from an independent experiment. (**b**) Skin-draining LN cells were incubated with 100 μM NiCl_2_ for 60 min with or without divalent cations at 500 μM. Results are shown as ΔMFI. Bars represent the mean ± SD of three independent experiments. Each symbol represents the value from an independent experiment. (**c**) Skin-draining LN cells were treated with trypsin for the indicated time, then incubated with 100 μM NiCl_2_ for 60 min followed by NPG. Results are shown as the ΔMFI of NPG, MFI of MHC class II and CD11c. Bars represent the mean ± SD of three independent experiments. Each symbol represents the value from an independent experiment. *P < 0.05, **P < 0.01, significantly different from the control (0 min). (**d**) Skin-draining LN cells were incubated with anti-MHC class II Abs for 20 min on ice, then incubated with 100 μM NiCl_2_ for 60 min followed by NPG. Results are shown as the ΔMFI of NPG. Bars represent the mean ± SD of three independent experiments. Each symbol represents the value from an independent experiment.

The biochemical properties of Ni-binding capabilities were also analyzed. ΔMFI was significantly decreased by the treatment with EDTA before NPG staining (Fig. 2a), indicating that Ni binding was reversible and removed by chelating. Whether divalent cations compete with the Ni binding was also analyzed. No significant changes in ΔMFI were detected in the presence of the other divalent cations, Ca^2+^, Mn^2+^,and Co^2+^ (Fig. 2b). Palladium ions show cross-reactivity with Ni^25^. However, due to high cytotoxicity, it was not possible to test Pd^2+^. ΔMFI was not decreased by trypsin-treatment (Fig. 2c), indicating that Ni-binding capabilities were trypsin resistant. The MFI of MHC class II increased significantly with trypsin treatment. On the other hand, the MFI of CD11c was trypsin-sensitive. We analyzed whether MHC class II is involved in the Ni-binding capabilities of migratory DCs in skin-draining LNs. Cells were preincubated with two kinds of anti-MHC class II mAbs, anti-I-A/I-E clone M5/114.15.2 and anti-I-A^b^ clone AF6-120.1, before incubation with Ni ions. However, neither of these Abs affected ΔMFI (Fig. 2d).

### Ni-binding capabilities differ between draining LNs

**Figure 3:**
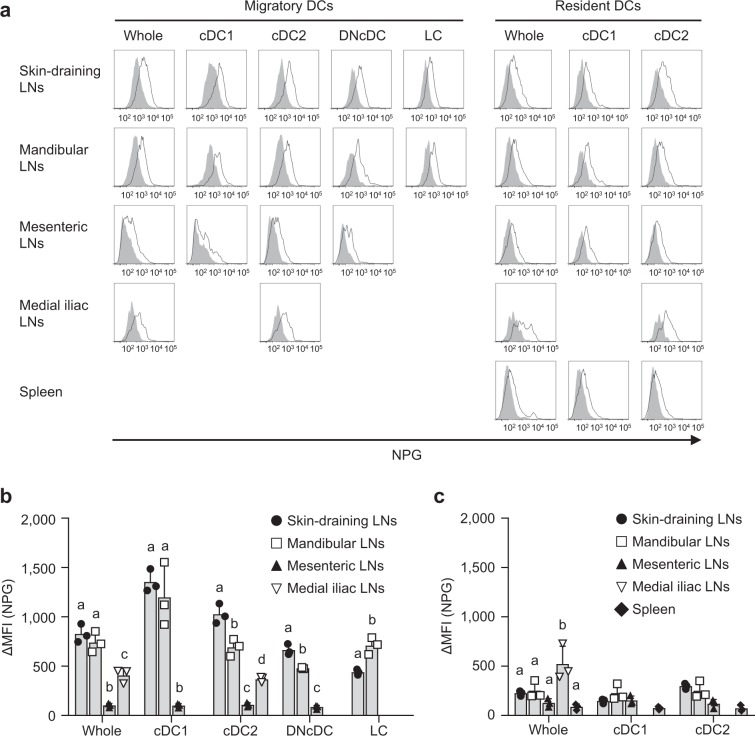
Ni-NPG staining of DCs from various LNs. (**a**) Cells were incubated with 100 μM NiCl_2_ for 60 min followed by NPG. Histograms represent the NPG fluorescence of cells incubated with (black line) or without (gray-shaded) NiCl_2_. Results are representative of three independent experiments. (**b** and **c**) Ni-NPG staining of migratory (**b**) and resident (**c**) DCs was analyzed. Results are shown as ΔMFI. Bars represent the mean ± SD of three independent experiments. Each symbol represents the value from an independent experiment. Means without a common letter were significantly different, *P* < 0.05.

Because Ni allergies mainly develop skin inflammation, we analyzed the Ni-binding capabilities of migratory and resident DCs in other LNs. Supplementary Fig. S2 shows the gating strategy of skin-draining, mandibular, mesenteric, and medial iliac LNs and the spleen. All LNs have migratory and resident DCs, whereas the spleen has only resident DCs. No XCR1^−^ EpCAM^+^ LC was detected in the migratory DCs of mesenteric and medial iliac LNs. Because of lower events, it was not possible to analyze XCR1^+^ cDC1 and XCR1^−^ EpCAM^−^ CD11b^−^ DNcDC in medial iliac LNs. All four subsets, XCR1^+^ cDC1, XCR1^−^ EpCAM^−^ CD11b^+^ cDC2, XCR1^−^ EpCAM^−^ CD11b^−^ DNcDC and XCR1^−^ EpCAM^+^ LC of migratory DCs exhibited strong Ni-binding capabilities in skin-draining and mandibular LNs (Fig. 3a,b). On the other hand, those in mesenteric LNs showed significantly weaker Ni-binding capabilities. The Ni-binding capabilities of migratory DCs in medial iliac LNs was significantly weaker than those of skin-draining and mandibular LNs, but significantly stronger than those of mesenteric LNs. Resident DCs in all LNs and the spleen exhibited weak Ni-binding capabilities (Fig. 3a,c). These results indicated that migratory DCs in skin-draining and mandibular LNs have strong Ni-binding capabilities.

### IL-1β induces MHC class II^hi^ cells with strong Ni-binding capabilities in ear pinnae

**Figure 4:**
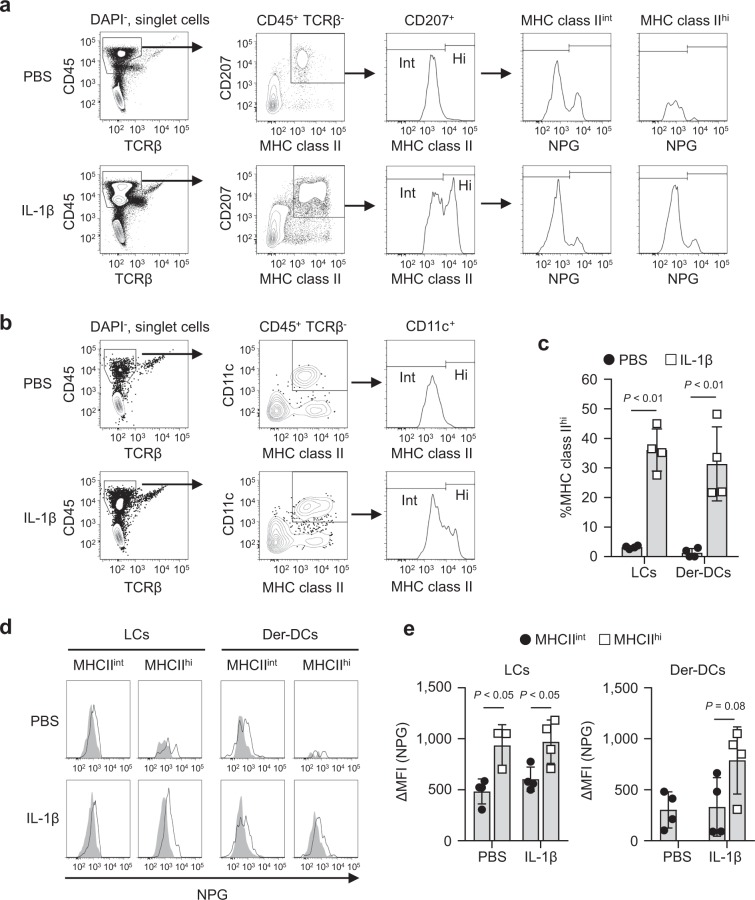
Ni-NPG staining of ear cells i.d. injected with IL-1β. Epidermal and dermal cells were prepared 24 h after injection with 50 ng of IL-1β or PBS. Cells were incubated with 100 μM NiCl_2_ for 60 min followed by NPG. (a and b) The gating strategies for epidermal (**a**) and dermal (**b**) cells are shown. (**c**) Percentage of MHC class II^hi^ cells in epidermal LCs and dermal DCs. Results represent the mean ± SD of four independent experiments. Each symbol represents the value from an independent experiment. (**d**) Histograms represent NPG fluorescence incubated with (black line) or without (gray-shaded) NiCl_2_. Results are representative of four independent experiments. (**e**) ΔMFI of epidermal LCs and dermal DCs. Results represent the mean ± SD of four independent experiments. Each symbol represents the value from independent experiment.

Ni-binding capabilities of APCs in local skin tissues were also analyzed. We hypothesized that inflammatory mediator(s) affects the Ni-binding capabilities. IL-1β is a critical inflammatory cytokine for Ni allergy and well-known activator of MHC class II^+^ APCs^24,26^. Therefore, we investigated the effects of IL-1β on the Ni-binding capabilities of APCs in ear pinnae. A flow cytometric analysis identified CD45^+^ CD207^+^ MHC class II^+^ epidermal LCs and CD45^+^ MHC class II^+^ CD11c^+^ dermal DCs in the epidermis and dermis, respectively (Fig. 4a,b). An i.d. injection of IL-1β significantly induced MHC class II^hi^ cells in LCs and dermal DCs. The percentage of MHC class II^hi^ cells was more than 10-fold higher in IL-1β-injected ear pinnae than in control PBS-injected ear pinnae (Fig. 4c). LCs divided NPG^lo^ and NPG^hi^, even in the absence of Ni, indicating that NPG^hi^ is non-specific NPG binding. Therefore, we examined the MFI of NPG^lo^ cells. Ni-binding capabilities were stronger in MHC class II^hi^ cells than in MHC class II^int^ cells, whereas no significant differences were observed between IL-1β- and PBS-injected ear pinnae (Fig. 4d,e). Because of low events, it was not possible to analyze the Ni-binding capabilities of MHC class II^hi^ dermal DCs in PBS-injected ear pinnae (Fig. 4e). These results indicated that IL-1β induces MHC class II^hi^ cells with strong Ni-binding capabilities in local ear pinnae.

### *In vivo* challenge with Ni induced Ni-binding LCs in draining LNs

**Figure 5:**
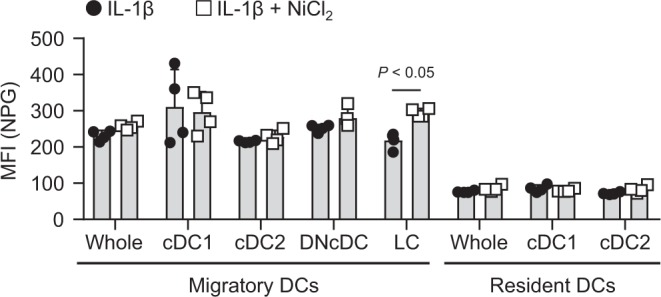
NPG staining of DCs in draining LNs after i.d. challenge with IL-1β and Ni. Ear pinnae of naïve mice were i.d. challenged 5 times every 24 h with 50 ng of IL-1β in the presence (left pinna) or absence (right pinna) of 10 mM NiCl_2_. Twenty-four hours after the last challenge, auricular LNs cells were stained with NPG followed by antibodies. Graph showing the MFI of NPG. Bars represent the mean ± SD of 4 pinnae from two independent experiments. Each symbol represents the value from each pinna.

We assessed whether an *in vivo* challenge induced Ni-binding DCs in draining LNs. Ear pinnae of naïve mice were i.d. challenged 5 times every 24 h with 50 ng of IL-1β in the presence or absence of 10 mM NiCl_2_. Twenty-four hours after the last challenge, cells from the draining LNs were stained with NPG in the absence of *in vitro* incubation with Ni. The MFI of NPG was significantly higher in LCs from the LNs challenged with IL-1β and Ni compared with IL-1β alone (Fig. 5). These results indicated that Ni-binding APCs were detected after an *in vivo* challenge with Ni.

### Induction of IL-1β in pan-DCs stimulated with Ni

**Figure 6:**
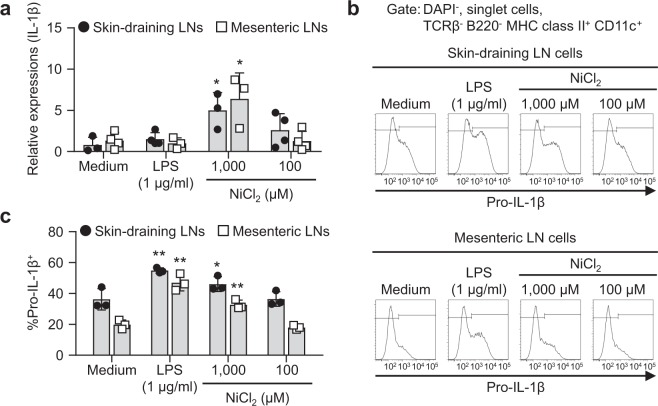
Induction of IL-1β in pan-DCs stimulated with Ni. (**a**) The pan-DC fraction from skin-draining or mesenteric LNs was incubated with LPS or NiCl_2_ for 4 h. The mRNA expression levels of IL-1β were analyzed by quantitative RT-PCR. Results represent the mean ± SD of four independent experiments. Each symbol represents the value from an independent experiment. *P < 0.05 significantly different from the medium. (**b**) Pan-DCs were incubated with LPS or NiCl_2_ for 4 h. After washing, cells were incubated further in the absence of any stimulation for 20 h. Intracellular pro-IL-1β was analyzed by flow cytometry. Representative results are shown. (**c**) The percentage of pro-IL-1β^+^ in MHC class II^+^ CD11c^+^ cells is shown. Bars represent the mean ± SD of three independent experiments. Each symbol represents the value from an independent experiment. *P < 0.05, **P < 0.01, significantly different from the medium.

We investigated whether Ni stimulation induced the expression of inflammatory cytokines. Due to the low cell number of DCs in LNs, we tried to expand the cell number of DCs *in vivo*. To achieve this, mice were s.c. injected B16 melanoma cells constitutively secreting Flt3 ligand (referred as B16-Flt3L) cells and LNs were collected 2 weeks after injection. Flt3L is a critical cytokine in DC-development^27^. Moreover, we sorted a pan-DC fraction from LNs and performed further experiments using these cells. The gating strategies of the skin-draining and mesenteric LN cells and pan-DC fraction from mice with B16-Flt3L are shown (Supplementary Figs. S3a–d). Ni-binding capabilities were stronger in migratory DCs of skin-draining LNs than other DCs in the same way as those from mice without B16-Flt3L (Supplementary Figs. S3e,f). Stimulation with 1,000 μM Ni for 4 h induced significantly higher expression levels of IL-1β mRNA in pan-DCs from skin-draining and mesenteric LNs (Fig. 6a). On the other hand, the mRNA expression levels of TNFα were increased significantly by stimulation with LPS but not Ni (Supplementary Fig. S4). Protein expression levels of intracellular pro-IL-1β were also analyzed. Cells were incubated with each stimuli for 4 h, then further incubated for 20 h without any stimulation. Significantly higher expression levels of intracellular pro-IL-1β were detected in MHC class II^+^ CD11c^+^ cells stimulated with 1,000 μM Ni or LPS for 24 h (Fig. 6b,c). These results indicated that Ni stimulation induced the expression of IL-1β.

### Ni-DCs elicit Ni-allergic inflammation

**Figure 7:**
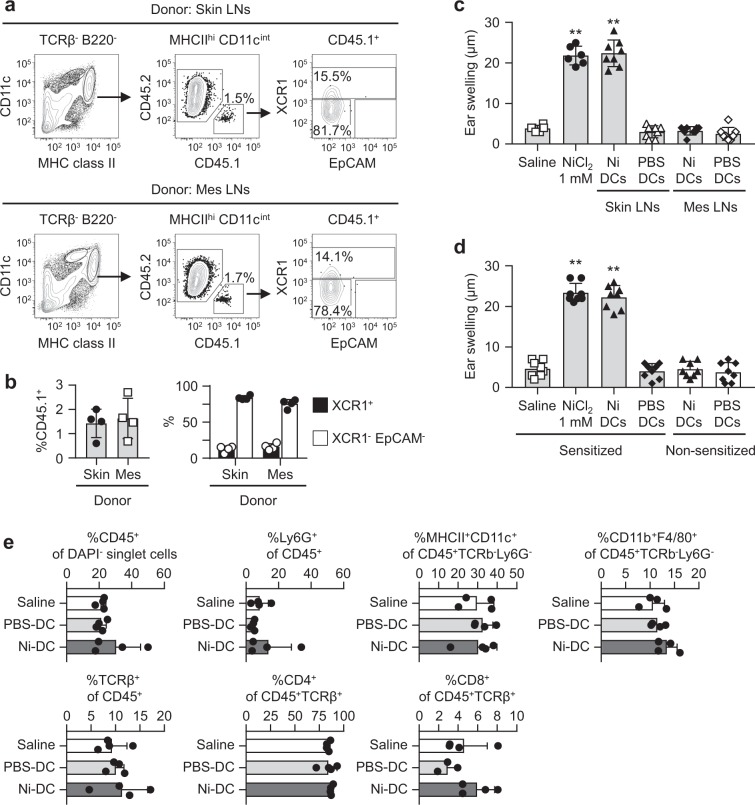
Elicitation of Ni-allergic inflammation by Ni-DCs. The pan-DC fraction was prepared from skin-draining or mesenteric LNs from CD45.1^+^ mice inoculated with B16-Flt3L. (**a**) A flow cytometric analysis of auricular LN cells from CD45.2^+^ recipient mice 24 h after i.d. injection of CD45.1^+^ pan-DCs (2 × 10^5^ cells) into the ear pinnae. Dot plots are representative of four mice. (**b**) Graphs showing %CD45.1^+^ in MHC class II^hi^ CD11c^int^ cells (left), %XCR1^+^, and %XCR1^-^ EpCAM^-^ in CD45.1^+^ cells (right). Bars represent the mean ± SD of four mice. Each symbol represents the value from each mouse. (**c**) The ear pinnae of Ni-sensitized mice were i.d. challenged with NiCl_2_ or DCs (1 × 10^4^ cells) prepared from skin-draining or mesenteric LNs. (**d**) The ear pinnae of Ni-sensitized and non-sensitized mice were i.d. challenged with NiCl_2_ or DCs (1 × 10^4^ cells) prepared from skin-draining LNs. Bars show ear swelling 48 h after the challenge. Results represent the mean ± SD of 6–8 pinnae (3–4 mice). Each symbol represents the value from each pinna. **P < 0.01, significantly different from the control (saline). (**e**) Ear pinnae of Ni-sensitized mice were i.d. challenged with NiCl_2_ or DCs (1 × 10^4^ cells) prepared from skin-draining LNs. Inflammatory cell recruitments into local ear tissues were analyzed 48 h after the challenge by flow cytometry. Bars represent the mean ± SD of 3–4 independent experiments. Each symbol represents the value from an independent experiment.

Finally, we examined whether Ni-binding DCs induce Ni-allergic inflammation. Pan-DCs were purified from skin-draining and mesenteric LNs of C57BL/6 CD45.1^+^ donor mice injected with B16-Flt3L, then i.d. injected into the ear pinnae of CD45.2^+^ recipient mice. CD45.1^+^ donor cells were detected in the migratory DC fraction of recipient’s auricular LNs 24 h after the i.d. injection, regardless of the origin of donor cells (Fig. 7a). Migratory DCs in skin-draining and mesenteric LNs of donor mice expressed a CCR7, a chemokine receptor that is responsible for the migration of DCs from local tissues to draining LNs (Supplementary Fig. S5a)^28^. CD45.1^+^ migratory DCs were subdivided as 15% XCR1^+^ and 80% XCR1^−^EpCAM^−^ cells. Percentages of CD45.1^+^ donor cells and those subsets were comparable in recipient mice that were injected with pan-DCs from the skin-draining and mesenteric LNs (Fig. 7b).

Pan-DCs were incubated with 100 μM Ni or PBS for 60 min (referred to as Ni-DCs and PBS-DCs, respectively), then i.d. injected into the ear pinnae of Ni-sensitized mice. Ni-DCs prepared from skin-draining LNs induced significantly greater ear swelling after i.d. injection (Fig. 7c and Supplementary Fig. S5b). However, the i.d. injection of Ni-DCs prepared from the mesenteric LNs did not induce ear swelling, even though donor cells were detected in the migratory DC fraction of auricular LNs in recipient mice (Fig. 7a–c). The i.d. injection of PBS-DCs prepared from the skin-draining and mesenteric LNs did not induced ear swelling. No ear swelling was induced in non-sensitized mice after i.d. injection with Ni-DCs prepared from skin-draining LNs (Fig. 7d), indicating that this ear swelling was an allergic, but not inflammatory, response. As reported previously^22^, no significant inflammatory cell migration was induced in our Ni allergy mouse model (Fig. 7e). We also investigated whether Ni-DCs induce sensitization; however, Ni sensitization was not induced by s.c. injection of Ni-DCs (Supplementary Fig. S5c). These results indicated that Ni-DCs induced Ni allergy at the elicitation phase.

## Discussion

To induce Ni-specific T cell activation, Ni ions need to be presented by professional APCs: however, the DC subsets responsible for this have not yet been identified. In the present study, we used an Ni-specific fluorescent probe NPG and clearly demonstrated that migratory DCs exhibited strong Ni-binding capabilities in skin-draining and mandibular LNs, but not in mesenteric or medial iliac LNs. An i.d. injection of IL-1β induced the activation of LCs and dermal DCs, which exhibited stronger Ni-binding capabilities. Ni-binding LCs were detected in draining LNs after i.d. challenge with IL-1β and Ni. Moreover, the i.d. injection of Ni-DCs prepared from skin-draining LNs, but not mesenteric LNs, elicited Ni-allergic inflammation. Previous studies reported that the human B cell line Raji cells showed Ni binding on the cell surface^23,29^. This is the first study to investigate the Ni-binding capabilities of DCs from various LNs.

Migratory DCs transport self and non-self Ags, which are captured in local tissues. On the other hand, resident DCs capture Ags that are incoming from blood and/or lymph flow^30^. Migratory DCs exhibited stronger Ni-binding capabilities than resident DCs in skin-draining and mandibular LNs. Skin and oral mucosa are considered to be the site at which Ni ions invade and are recognized by the host immune system. Ni-binding capabilities were significantly weaker in mesenteric and medial iliac LNs. It was reported that even contacts with Ni through intestinal mucosa by the ingestion of Ni-containing foods induce Ni-allergic dermatitis, also known as systemic contact dermatitis^31^. These results suggested that the Ni-binding capabilities of migratory DCs in draining LNs are important for establishing whether tissues have Ni-allergic responses.

We used 100 μM NiCl_2_ for *in vitro* incubation with cells. Tanaka *et al*. reported that the elution of Ni ions from a metal wire was enhanced by inflammation^32^. They reported that approximately 100 nmol/g tissue of Ni ions was eluted from the metal wire implanted subcutaneously into the dorsum with injection of LPS into the site immediately after implantation. Murine macrophages stimulated with LPS eluted approximately 250 μM Ni ions from the Ni plate. Therefore, we considered that the concentration (100 μM) of NiCl_2_ used in this study was suitable for the site of local inflammation.

Although we did not identify Ni-binding receptor(s) on migratory DCs, we noted some biochemical properties. Ni-binding capabilities were trypsin-resistant, reversible, and did not compete with other divalent cations, such as Ca^2+^, Mn^2+^, and Co^2+^. These results suggested that Ni binds to specific receptor(s), but not to general metal-binding sites.

Preincubation with anti-MHC class II mAbs did not affect ΔMFI. On the other hand, Ni-binding capabilities were significantly stronger in MHC class II^hi^ cells. Not only the Ni-binding capabilities but also the expression levels of MHC class II were higher in migratory DCs in skin-draining and mandibular LNs than those in mesenteric LNs. MHC class II^hi^ cells in local skin tissues showed stronger Ni-binding capabilities. Ags captured at local sites and presented on MHC molecules may be responsible for Ni-binding capabilities. However, histogram analysis showed that incubation with Ni induced a peak shift but not a NPG^hi^ subpopulation. These results indicated that all migratory DCs, but not specific subpopulation(s), have Ni-binding capabilities. Further studies are needed to clarify whether MHC class II and Ag peptides on MHC molecules are involved in Ni-binding capabilities.

XCR1^+^ cDC1s exhibited strong Ni-binding capabilities. cDC1 has cross-presentation activities and activated CD8^+^ T cells^4^. CD8^+^ T cells play important roles in Pd allergy^12^, suggesting that the stronger Ni-binding capabilities of cDC1s effectively activated Ni-specific CD8^+^ T cells. XCR1^-^ cD11b^+^ cDC2s also exhibited strong Ni-binding capabilities. cDC2s are major dermal DCs that migrate and transport Ags to draining LNs^33^. XCR1^-^ CD11b^-^ DNcDCs are activated by TSLP in contact hypersensitivity^6^. Although TSLP induces Th2 cytokines, it plays a role in Th1-type Ni allergy^15^. DNcDCs with Ni-binding capabilities may contribute to the TSLP-dependent pathway of Ni allergy. LCs in skin-draining LNs also exhibited Ni-binding capabilities. Moreover, Ni-binding LCs were detected in draining LNs after *in vivo* challenge with IL-1β and Ni. LCs have been reported to induce Th17 responses^3^. Analyses with human samples revealed that Th17 were also induced in Ni allergy^14^. The Ni-binding capabilities of LCs may be involved in the induction of Th17.

An i.d. injection of IL-1β into ear pinnae activated epidermal LCs and dermal DCs with high expression levels of MHC class II. These MHC class II^hi^-LCs and dermal DCs exhibited stronger Ni-binding capabilities than non-activated, MHC class II^int^ cells. In addition, the i.d. injection of IL-1β and Ni into ear pinnae induced Ni-binding LCs in draining LNs. IL-1 is a critical inflammatory cytokine of contact hypersensitivity^34,35^. The Ni allergy mouse model used in this study depends on IL-1^24^. We reported previously that the adjuvant activity of resin monomers in Ni allergy was dependent on IL-1^36^. These findings suggested that the activation of LCs and dermal DCs with strong Ni-binding capabilities is one of the pathological roles of IL-1β in Ni allergy.

Stimulation with Ni induced the expression of IL-1 β mRNA in the pan-DC fraction from not only skin-draining LNs but also mesenteric LNs. Intracellular pro-IL-1β was also induced by Ni stimulation in skin-draining and mesenteric LN cells. This induction was only detected at a higher concentration (1,000 μM) of Ni. However, 100 μM Ni, a concentration that induced significant Ni binding in skin-draining LN cells, did not induce the expression of IL-1β. These results suggested that the expression of IL-1β is not induced by Ni binding. Ni activates some transcriptional factors that contribute to the expression of IL-1β^37–39^. Moreover, a Ni stimulation has been shown to activate MAP kinase kinase 6 in murine DCs^20^. Further studies are needed to clarify the induction mechanisms of IL-1β in Ni-stimulated DCs.

Donor cells were detected in auricular LNs of recipient mice after an i.d. injection of pan-DCs prepared from skin-draining and mesenteric LNs. We detected the expression of CCR7 on migratory DCs in skin-draining and mesenteric LNs. CCR7 is important for the migration of DCs to draining LNs from local tissues such as skin and gut^28^. Significant ear swelling was induced after the i.d. injection of Ni-DCs prepared from skin-draining LNs, but not those from mesenteric LNs, into the ear pinnae of Ni-sensitized mice. These results suggested that DCs with strong Ni-binding capabilities elicited Ni-allergic inflammation. A previous study reported that migratory DCs, particularly CD103^−^ cDC2s, have a short lifespan and die by apoptosis 2 d after migration^33^. Ags carried by migratory DCs are transferred to and presented by resident DCs^40^. Therefore, Ni-DCs may also die after migrating to draining LNs. Further studies are needed to clarify whether Ni-DCs directly activate Ni-specific T cells.

Our present results suggest novel therapeutic strategies for Ni allergy. Ni-binding receptor(s) may be a potential therapeutic target. Functional manipulation of Ni-binding DCs is also possible therapeutic approach. Moreover, appropriate targeting of migratory cDC2 in skin-draining and mandibular LNs may be useful for Ni-specific immunotherapy. Dermal CD11b^+^ cDCs show aldehyde dehydrogenase activity, which is involved in retinoic acid production, and induce Foxp3^+^ Treg cells^41^. Retinoic acid is required the *de novo* generation of Foxp3^+^ Treg cells^8^. Foxp3^-^ latency-associated peptide^+^ Treg cells are also induced by dermal CD11b^+^ cDC2s^42^. Oral CD11b^+^ cDCs also show aldehyde dehydrogenase activity and induce Foxp3^+^ Treg cells in mandibular LNs^9^. These findings suggested that Ni-binding cDC2s in the skin-draining and mandibular LNs can induce Ni-specific Treg cells.

In conclusion, the present study clearly demonstrated that migratory DCs in skin-draining and mandibular LNs exhibited Ni-binding capabilities. Ni-treated DCs purified from skin-draining LNs elicited Ni-allergic inflammation. However, there are still unresolved questions regarding Ni-binding receptor(s) and the mechanisms by which Ni-DCs elicit Ni-allergic inflammation. The elucidation of these questions will be of value for the development of effective methods to prevent and treat metal allergies.

## Materials and Methods

### Mice

Female C57BL/6 N CD45.2^+^ mice were purchased from CLEA Japan (Tokyo, Japan). Congenic C57BL/6 CD45.1^+^ mice were provided by the RIKEN BRC through the National Bio-Resource Project of the MEXT, Japan. The nomenclature of LNs was in accordance with that reported previously^43^. Superficial parotid (auricular), proper axillary, accessory axillary, and subiliac (inguinal) LNs were pooled and used as skin-draining LNs. Jejunal LNs were used as mesenteric LNs. All animal procedures were approved by the Institutional Animal Care and Use Committee of Tohoku University (approved number: 2016DnA-054) and the Genetic Modification Safety Committee of Tohoku University (approved number: 2016DnLMO-020), and were performed in accordance with their guidelines.

### LN cell preparation

LNs were minced and incubated with 1 mg/ml collagenase D (Roche, Basel, Switzerland) and 5 μg/ml DNase (SIGMA-Aldrich, St Louis, MO) in DMEM containing 10% FCS at 37 °C for 30 min with shaking. Cells were filtered using a 70-μm cell strainer.

To analyze draining LNs after *in vivo* challenge, ear pinnae of naïve mice were i.d. challenged (20 μl/ear) 5 times every 24 h with 50 ng of IL-1β (BioLegend, San Diego, CA) in the presence (left pinna) or absence (right pinna) of 10 mM NiCl_2_. Twenty-four hours after the last challenge, auricular LNs were harvested, and LN cells were prepared without enzymatic treatment.

### Ear cell preparation

Ear pinnae were i.d. injected (20 μl/ear) with 50 ng of IL-1β or PBS containing 2.5% FCS and collected 24 h after the injection. Ear pinnae were split along with cartilage and incubated with 0.25% trypsin and 1 mM EDTA (Nacalai Tesque, Kyoto, Japan) at 37 °C for 1 h. After separating the epidermis and dermis, epidermal cells were prepared by pipetting. The dermis was minced and incubated with 1 mg/ml type-IV collagenase (SIGMA-Aldrich) and 0.1 mg/ml DNase in DMEM containing 10% FCS at 37 °C for 2 h with shaking. Cells were filtered using a 70-μm cell strainer. To analyze inflammatory cell infiltration, ear pinnae from the Ni allergy mouse were minced and incubated with 1 mg/ml type-IV collagenase and 0.1 mg/ml DNase in DMEM containing 10% FCS at 37 °C for 2 h with shaking. Cells were filtered using a 70-μm cell strainer.

### Flow cytometry

Cells were incubated with 100 μl of NiCl_2_ in PBS containing 0.01% FCS at 37 °C for the indicated time. After washing with PBS, cells were further incubated with 100 μl of NPG-DCF dipotassium salt (1 μM in PBS, Thermo Fisher Scientific, Waltham, MA) for 30 min. In some experiments, cells were treated with 1 mM EDTA-PBS at 37 °C for 30 min before staining with NPG. Cells were incubated with anti-CD16/32 (2.4G2, produced in-house) to block of non-specific binding, then stained with antibodies. The antibodies used were listed in Supplementary Table S1. To analyze the neutralizing effects of anti-MHC class II Abs, cells were preincubated with antibodies for 20 min on ice before incubation with Ni and NPG. Regarding intracellular cytokine staining, cells were fixed with Cytofix/Cytoperm (BD Biosciences, San Jose, CA) and washed with Perm Wash Buffer (BioLegend). Dead cells were stained with DAPI for extracellular staining or Zombie Yellow (BioLegend) for intracellular cytokine staining and excluded. Data were acquired on an LSRFortessa cell analyzer (BD Biosciences) and analyzed using FlowJo software (Tree Star, Ashland, OR). Geometrical mean fluorescence intensity was shown as mean fluorescence intensity (MFI). The results of Ni-NPG staining are shown as delta MFI (ΔMFI), calculated as [MFI^Ni 100 μM^ – MFI^Ni 0 μM^].

### Trypsin treatment of LN cells

LN cells were incubated with 100 μl of 0.25% Trypsin-1 mM EDTA solution for the indicated time. To stop trypsin digestion, 1 ml of 10% FCS-PBS was added. After incubation on ice for 5 min, the cells were stained for flow cytometric analyses.

### *In vivo* FMS-like tyrosine kinase 3 ligand treatment

To expand DCs *in vivo*, mice were s.c. injected B16-Flt3L, which were provided by Dr. A. Kumanogoh (Osaka University, Osaka, Japan). Flt3L is a cytokine that induces DC development^27^. B16-Flt3L cells were grown in DMEM containing 10% FCS. A total of 2 × 10^6^ cells/200 μl were s.c. injected into the dorsal region of mice. Mice were killed and tissues were collected 2 weeks after the injection.

### Pan-DC enrichment

The pan-DC fraction was prepared by negative selection using the EasySep Mouse Pan-DC Enrichment Kit (STEMCELL Technologies, Vancouver, BC, Canada) as described in the instruction manual.

### *In vitro* stimulation assay

Cells (1 × 10^6^ cells/ml) were suspended in RPMI1640 containing 10% FCS, 1 mM sodium pyruvate and 50 μM 2-mercaptoethanol and stimulated with LPS (1 μg/ml) or NiCl_2_ (1,000 or 100 μM) for 4 h. Cells were collected and analyzed by quantitative RT-PCR. In intracellular cytokine staining, cells were washed and incubated further without any stimulation for 20 h.

### Quantitative RT-PCR

Cells were homogenized in ISOGEN II (Nippon Gene, Tokyo, Japan) and total RNA was extracted as described by the manufacturer. Complement DNA (cDNA) was synthesized using a Transcriptor First Strand cDNA Synthesis Kit (Roche). Quantitative RT-PCR was performed using the SYBR Select Master Mix and StepOnePlus realtime PCR system (Applied Biosystems, Waltham, MA). The primers used for quantitative RT-PCR were listed in Supplementary Table S2. mRNA expression levels were calculated by the ΔΔCT method using β-actin as an inner control.

### Ni allergy mouse model

Pan-DCs (1 × 10^6^ cells) were incubated with 100 μl of 100 μM NiCl_2_ in PBS containing 0.01% FCS at 37 °C for 60 min. After washing with PBS, the cells were suspended in saline and referred to as Ni-DCs. The pan-DCs incubated without Ni were referred to as PBS-DCs. A Ni allergy mouse model was constructed as previously reported^24^. Briefly, mice were sensitized with an intraperitoneal (i.p.) injection of solution containing 0.5 mM NiCl_2_ and 0.5 μg/ml LPS. Two weeks after sensitization, mice were challenged with 20 μl of NiCl_2_ or the pan-DC fraction by an i.d. injection into both pinnae. Ear swelling was measured 48 h after the challenge using the Peacock dial thickness gauge. Inflammatory cell migration was analyzed by flow cytometry.

### Statistical analysis

Experimental values were given as the mean ± SD. Statistical analyses were performed with a one- or two-way ANOVA using Tukey’s, Dunnett’s, and Sidak’s multiple comparisons tests and an unpaired two-tailed t-test (GraphPad Prism version 7.02, GraphPad Software, San Diego, CA). *P* < 0.05 was considered to be significant. Data shown are representative of at least two independent experiments.

## Supplementary information

Supplementary information.

## Data Availability

The datasets generated during and/or analyzed during the current study are available from the corresponding author on reasonable request.
