# Supplementary material for: Migratory dendritic cells in skin-draining lymph nodes have nickel-binding capabilities
Source: Sci Rep. 2020 Mar 19;10:5050. doi: 10.1038/s41598-020-61875-6 (PMC7081353; doi:10.1038/s41598-020-61875-6)
Supplement: Supplementary file 1 — Supplementary information. [file 41598_2020_61875_MOESM1_ESM.pdf]

Migratory dendritic cells in skin-draining lymph nodes have nickel-binding capabilities

Toshinobu KUROISHI, Kanan BANDO, Reiska Kumala BAKTI, Gaku OUCHI, Yukinori TANAKA, and Shunji SUGAWARA

Supplementary Table S1. Antibodies used for flow cytometry

Antigen	Clone	Fluorochrome	Manufacturer
B220 (CD45R)	RA3-6B2	PE	BioLegend
		PE/Cy5	BioLegend
CCR7	4B12	APC	BioLegend
CD103	2E7	APC	BioLegend
CD11b	M1/70	PerCP/Cy5.5	BioLegend
		APC/Cy7	BioLegend
CD11c	N418	PE/Cy7	BioLegend
CD19	1D3	PE/Cy5	BioLegend
CD4	RM4-5	APC/Cy7	BioLegend
CD45	30-F11	PerCP	BioLegend
CD45.1	A20	Brilliant Violet 421	BioLegend
CD45.2	104	FITC	BioLegend
CD207 (langerin)	4C7	PE	BioLegend
CD8α	53-6.7	Alexa Fluor 647	BioLegend
EpCAM (CD326)	G8.8	APC/Cy7	BioLegend
F4/80	BM8	APC	BioLegend
MHC class II (I-Ab)	AF6-120.1	Pacific blue	BioLegend
MHC class II (I-A/I-E)	M5/114.15.2	Pacific blue	BioLegend
		PE/Cy7	
TCRβ	H57-597	PE, PE/Cy5	BioLegend

PDCA1 (CD317)	129C1	Alexa Fluor 647	BioLegend
XCR1	ZET	APC	BioLegend
Pro-IL-1β	NJTEN-3	PE	eBioscience

Supplementary Table S2. Primers used for quantitative RT-PCR

Target (Accession number)		Sequence
Mouse IL-1β (M15131)	Forward	TTCAGGCAGGCAGTATCA
	Reverse	CCAGCAGGTTATCATCATCATC
Mouse TNFα (M13049)	Forward	AGCCTCTTCTCATTCCTGC
	Reverse	GGAGGCCATTTGGGAACT
Mouse β-actin (NM_007393)	Forward	CGTTGACATCCGTAAAGACCTC
	Reverse	AGCCACCGATCCACACAGA

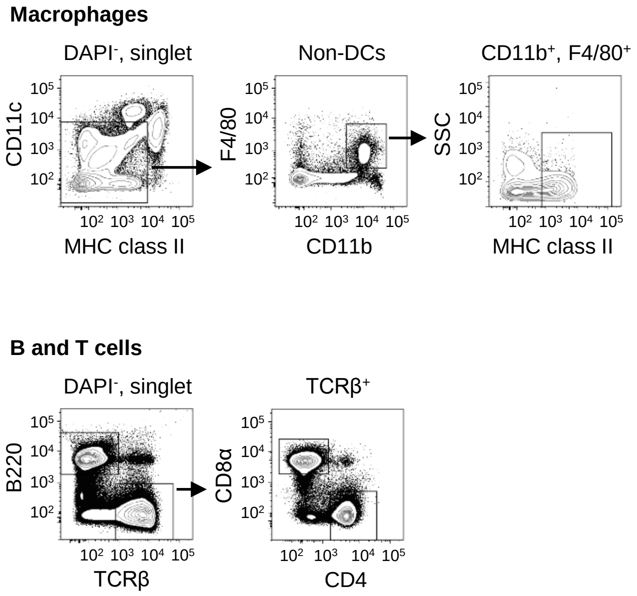

Figure S1. Gating strategies of macrophages (non-DCs $CD11b^{+}F4/80^{+}$MHC class II^{+}), $B220^{+}$ B cells, and $CD4^{+}$ and $CD8\alpha^{+}$ T cells are shown.

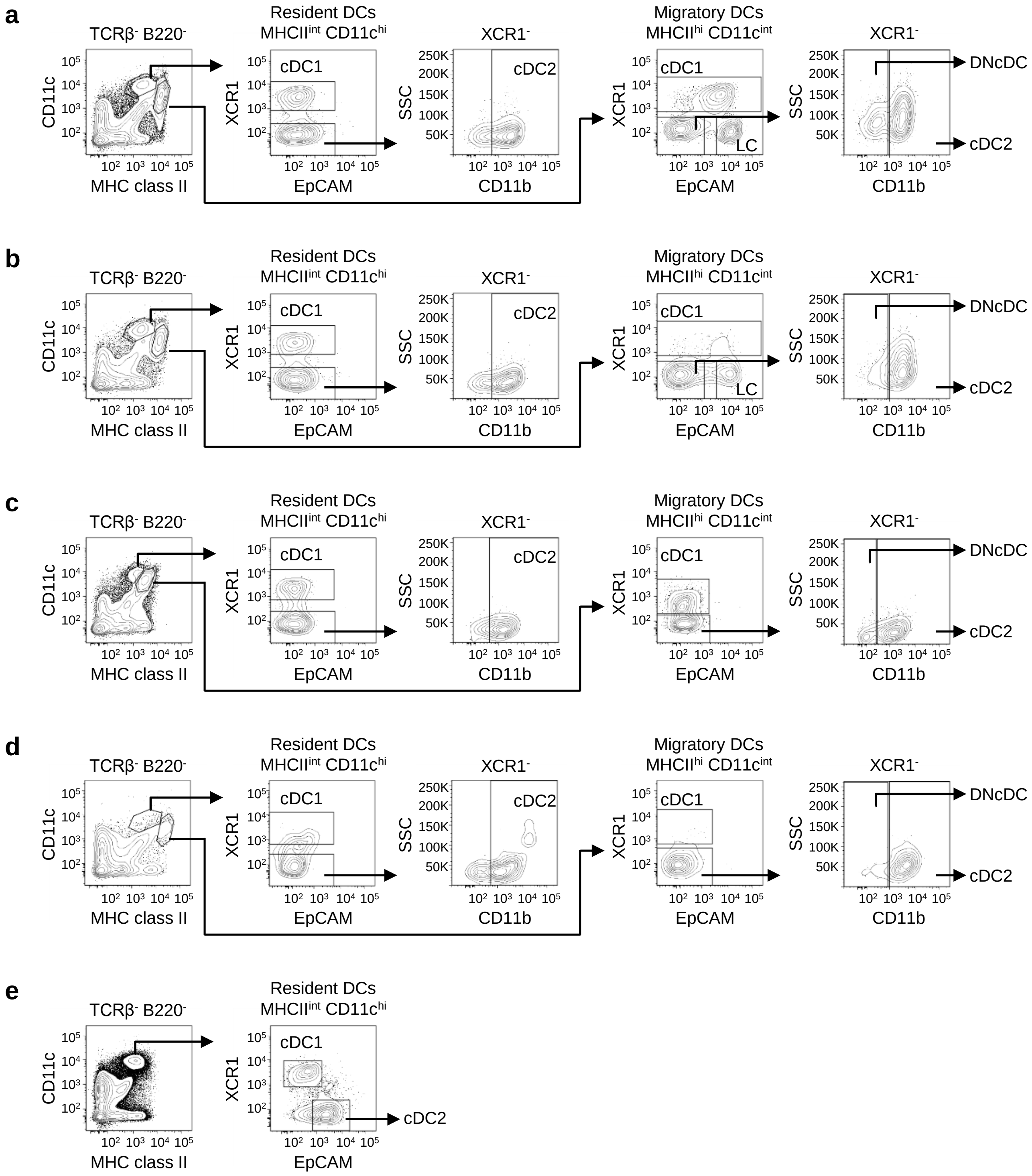

Figure S2. Gating strategies of skin-draining (a), mandibular (b), mesenteric (c), and medial iliac (d) LNs, and the spleen (e) are shown.

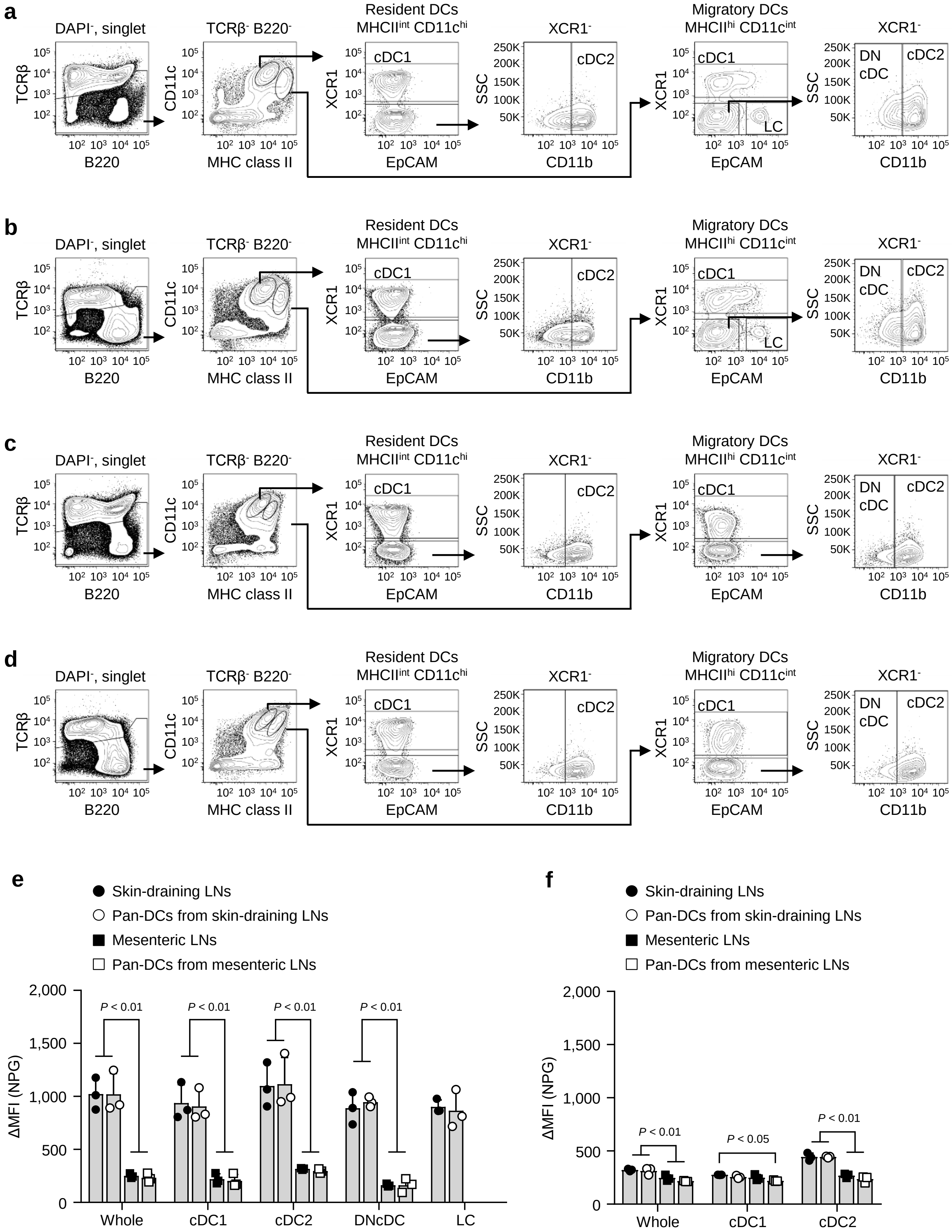

Figure S3. Ni-NPG staining of skin-draining and mesenteric LN cells and pan-DCs from mice inoculated with B16-Flt3L. (a-d) The gating strategy is shown. a, skin-draining LN cells; b, pan-DCs from skin-draining LNs; c, mesenteric LN cells; d, pan-DCs from mesenteric LNs. (e and f) Cells were incubated with 100 μM $NiCl_2$ for 60 min followed by NPG. The Ni-NPG staining of migratory (e) and resident (f) DCs was analyzed. Results are shown as ΔMFI. Bars represent the mean ± SD of 3 mice. Each symbol represents the value from each mouse.

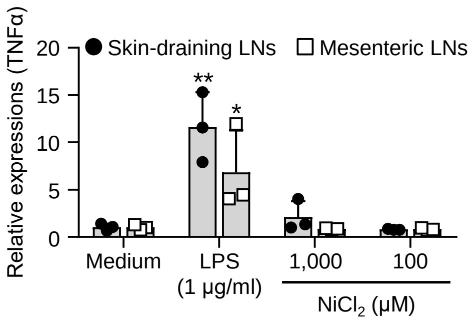

Figure S4. The pan-DC fraction from skin-draining or mesenteric LNs was incubated with LPS or $NiCl_2$ for 4 h. The mRNA expression levels of TNFα were analyzed by quantitative RT-PCR. Results represent the mean ± SD of three independent experiments. Each symbol represents the value from an independent experiment. *$P < 0.05$, **$P < 0.01$, significantly different from the medium.

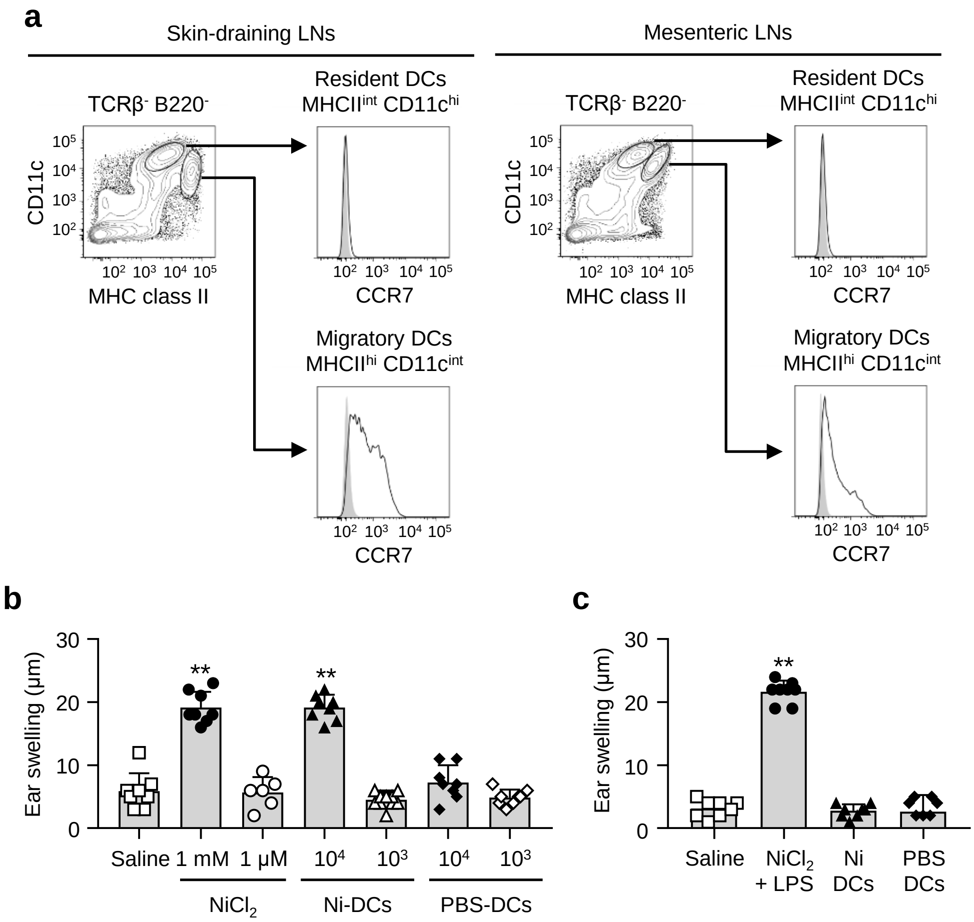

Figure S5. (a) Histograms represent CCR7 expression of migratory and resident DCs in skin-draining and mesenteric LNs from mice inoculated with B16-Flt3L (black line: anti-CCR7, gray-shaded: fluorescence minus one). Results are representative of three mice. (b) The ear pinnae of Ni-sensitized mice were i.d. challenged with $NiCl_2$ or DCs prepared from skin-draining LNs. (c) Mice were i.p. sensitized with 0.5 mM $NiCl_2$ and 0.5 μg/ml LPS or s.c. sensitized with DCs (1×10^5 cells). Two weeks after sensitization, the ear pinnae were i.d. challenged with 1 mM $NiCl_2$. Bars show ear swelling 48 h after the challenge. Results represent the mean ± SD of 6-8 pinnae (3-4 mice). Each symbol represents the value from each pinna. $^{**}P < 0.01$, significantly different from the control (saline).
